# Collagen type XVIII alpha 1 chain (*COL18A1*) variants affect the risk of anti‐tuberculosis drug‐induced hepatotoxicity: A prospective study

**DOI:** 10.1002/jcla.23630

**Published:** 2020-12-09

**Authors:** Yuhui Cheng, Lin Jiao, Weixiu Li, Jialing Wang, Zhangyu Lin, Hongli Lai, Binwu Ying

**Affiliations:** ^1^ West China School of Medicine Sichuan University Chengdu China; ^2^ Department of Laboratory Medicine West China Hospital Sichuan University Chengdu China

**Keywords:** anti‐tuberculosis drug‐induced hepatotoxicity, *COL18A1*, genetic polymorphisms, susceptibility

## Abstract

**Background:**

The role of collagen type XVIII alpha 1 chain (*COL18A1*) in anti‐tuberculosis drug‐induced hepatotoxicity (ATDH) has not been reported. This study aimed to explore the association between of *COL18A1* variants and ATDH susceptibility.

**Methods:**

A total of 746 patients were enrolled in our study from December 2016 to April 2018, and all subjects in the study signed an informed consent form. The custom‐by‐design 2x48‐Plex SNPscanTM kit was used to genotype all selected 11 SNPs. Categorical variables were compared by chi‐square (χ^2^) or Fisher's exact test, while continuous variables were compared by Mann‐Whitney's U test. Plink was utilized to analyze allelic and genotypic frequencies, and genetic models. Multivariate logistic regression analyses were used to adjust potential factors. The odds ratios (ORs) with corresponding 95% confidence intervals (CIs) were also calculated.

**Results:**

Among patients with successfully genotyping, there were 114 cases and 612 controls. The mutant A allele of rs12483377 conferred the decreased risk of ATDH (OR = 0.13, 95%CI: 0.02–0.98, *P* = 0.020), and this significance still existed after adjusting age and gender (*P* = 0.024). The mutant homozygote AA genotype of rs12483377 was associated with decreased total protein levels (*P* = 0.018).

**Conclusion:**

Our study first revealed that the A allele of *COL18A1* rs12483377 was associated with the decreased risk of ATDH in the Western Chinese Han population, providing new perspective for the molecular prediction, precise diagnosis, and individual treatment of ATDH.

## Graphical abstract

Regarding the heavy burden of ATDH in Western China, we thus conducted a prospective study to explore the association between COL18A1 polymorphisms and the risk of ATDH in the Western Chinese Han population. In this work, we first demonstrated the relationship between *COL18A1* rs12483377 and ATDH and put forward the possible function mechanisms, contributing to further understanding of the molecular pathogenesis of ATDH and promoting the development of molecular diagnostics.

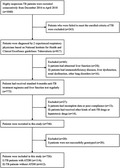

## 1 INTRODUCTION

Anti‐tuberculosis drug‐induced hepatotoxicity (ATDH) is the most serious adverse drug reaction during the course of tuberculosis (TB) therapy.^1^ ATDH is defined as the inflammation of hepatocytes caused by idiosyncratic reaction to the anti‐TB drugs.^2^ The following 4 mechanisms are considered as the pathogenesis of ATDH: I) the enzymes and pathways about drug metabolizing, such as glutathione S‐transferase (GST) and *N*‐acetyl transferase 2 (NAT2); II) the accumulation of bile acids, lipids, and heme metabolites; III) the toxicity mediated by immune system; IV) the increasing oxidant stress.^3^, ^4^, ^5^, ^6^ It is noted that ATDH can be curable in the early stage,^7^ although this disease has high mortality (22.7%) and morbidity (28%) ^8^, ^9^, ^10^ and adverse effects on the anti‐TB treatment efficiency.^11^ However, the ambiguous diagnostic criteria and atypical symptoms interfere with early prediction and diagnosis of ATDH. Even worse, the delayed diagnosis of ATDH aggravates the severity of the disease and increases the disease burden.^12^, ^13^ Clearly, it is urgent to explore new biomarkers for diagnosis of ATDH. With the development of molecular detection methods, genetic factors are gradually well‐recognized and considered as the crucial elements in the pathogenesis, prediction, diagnosis and treatment of many diseases.^14^, ^15^, ^16^ Nowadays, a growing body of evidence proves that single nucleotide polymorphisms (SNPs), such as pregnane X receptor (*PXR*) rs7643645^17^, ^18^ and phase I cytochrome P450 enzyme (*CYP2E1*),^8^ play important roles in the prediction, diagnosis, and treatment of ATDH. However, these SNPs are not applied in clinic due to limited predictive capacity or reliability. Thus, there is a promising future for exploring the association between more meaningful SNPs and ATDH to achieve precise prediction and treatment of ATDH.

Collagen type XVIII alpha 1 chain (*COL18A1*) is located on chromosome 21q22.3, encoding the alpha XVIII collagen. The product of alpha XVIII collagen, endostatin (EST), is mainly present in the liver sinusoidal and basement.^19^ The close relationship between EST and liver diseases has been reported in many studies.^20^, ^21^, ^22^ Many researchers find that EST can initiate the nicotinamide adenine dinucleotide phosphate oxidase (NOX) redox signaling cascade.^20^, ^23^, ^24^ While the activation of NOX can generate reactive oxygen species (ROS) to increase oxidant stress which is one of the pathogenesis of ATDH as described before, and thus leads to the exacerbation of liver injury.^4^, ^23^, ^25^ Moreover, Wnt/β‐catenin signaling directs multiple liver cell processes and it is the essential signal for protecting hepatocyte from oxidative stress‐induced cell deaths.^26^ Moreover, it is has been published that EST can inhibit Wnt/β‐catenin signaling through promoting the degradation of β‐catenin.^27^ Hence, we speculated that *COL18A1* plays a role in ATDH by involving in the Wnt/β‐catenin signaling, oxidative stress, and other various ways.^28^, ^29^, ^30^

It is a pity that few studies have paid attention to explore the correlation between *COL18A1* and ATDH. Regarding the high burden of ATDH in Western China,^31^ we conducted this prospective study to evaluate the association between *COL18A1* polymorphisms and the risk of ATDH in the Western Chinese Han population for the first time. We aimed to explore novel targets for the pathogenesis and personal treatment of ATDH patients.

## 2 METHODS

### 2.1 Study population

In this prospective study, 746 subjects were recruited from the West China Hospital of Sichuan University from December 2016 to April 2018 consecutively. All enrolled patients in the study were unrelated Han ethnicity.

The study was approved by the Ethics Committee of West China Hospital of Sichuan University (Reference No. 198; 2014), and written informed consents were obtained from all patients.

### 2.2 Inclusion criteria and exclusion criteria

All recruited patients must meet the following criteria: I) Patients were newly diagnosed as TB patients by 2 experienced respiratory physicians based on National Institute for Heath and Clinical Excellence (NICE) guidelines [NG33]: Tuberculosis ^32^; II) patients should have normal liver function before the anti‐TB treatment. If patients who had (a) immunodeficiency diseases such as HIV; (b) liver dysfunction such as hepatitis B or C infection, fatty liver; (c) received other hepatotoxic drugs; (d) renal dysfunction; (e) other lung or liver disorders such as lung cancer and cirrhosis would be excluded.

Included subjects would be treated with the WHO standard 6‐months anti‐TB treatment regimens, consisting of isoniazid (INH), rifampicin (RIF), pyrazinamide (PZA), and ethambutol (EMB). Besides, enrolled subjects did not take any other drugs which would cause liver damage.

During the anti‐TB therapy, patients would be tested liver function regularly to monitor their liver function and the baseline levels of laboratory indicators before anti‐TB treatment were tested. ATDH was defined as follows ^33^: (a) an increase in alanine aminotransferase (ALT) levels more than 2 times upper limit of normal (ULN); (b) an increase in ALT 2 times upper ULN combined a rise in aspartate aminotransferase (AST) or total bilirubin (TB) levels.

### 2.3 SNP selection

The genetic data of *COL18A1* were obtained from 1000 Genomes database. All SNPs should meet the criteria that the minor allele frequency (MAF) was greater than 0.02. The tag SNPs selected by Haploview (version 4.1), the locations of SNPs and relevant reports^34^, ^35^, ^36^, ^37^, ^38^, ^39^, ^40^, ^41^ also needed to be considered. Eventually, 11 SNPs (rs2236455, rs114220916, rs9980080, rs2236467, rs13048803, rs2838942, rs9980525, rs3753019, rs2236483, rs12483377, rs7867) were chosen.

### 2.4 Genotyping

Peripheral whole blood specimens were collected from each enrolled patient. All these samples were used to extract genomic deoxyribonucleic acid (DNA) via QLAamp^®^ DNA Blood Mini Kit (Qiagen, Germany). Then, the custom‐by‐design 2x48‐Plex SNPscanTM kit (Genesky Biotechnologies Inc, Shanghai, China) was utilized for genotyping all SNPs. All processes were carried out in accordance with the instructions.

### 2.5 Statistical analysis

Categorical variables such as gender and drinking statuses were compared by chi‐square (χ^2^) or Fisher's exact test, whereas continuous variables such as age and serum ALT levels were compared by Mann‐Whitney's U test. The Hardy‐Weinberg equilibrium (HWE), allelic and genotypic frequencies, and genetic models (addictive, dominant and recessive model) were all performed by Plink (version 1.07). Multivariate logistic regression analyses were used to adjust potential impact factors via SPSS (IBM, Chicago, IL, USA; Version 22.0). Linkage disequilibrium (LD) and haplotype association were analyzed by both Haploview (The Broad Institute, Cambridge, MA, USA; Version 4.1) and online tool SNPstats (https://www.snpstats.net/preproc.php). Odds ratios (ORs) and 95% confidence intervals (CIs) were calculated for correlations. *P* valve ≤ 0.05 was considered to be statistically significant. Statistical Power was calculated by Power and Sample Size Program software. Ordinary one‐way ANOVA was conducted by GraphPad Prism (version 8.0).

## 3 RESULTS

### 3.1 Study characteristics

**Figure 1:**
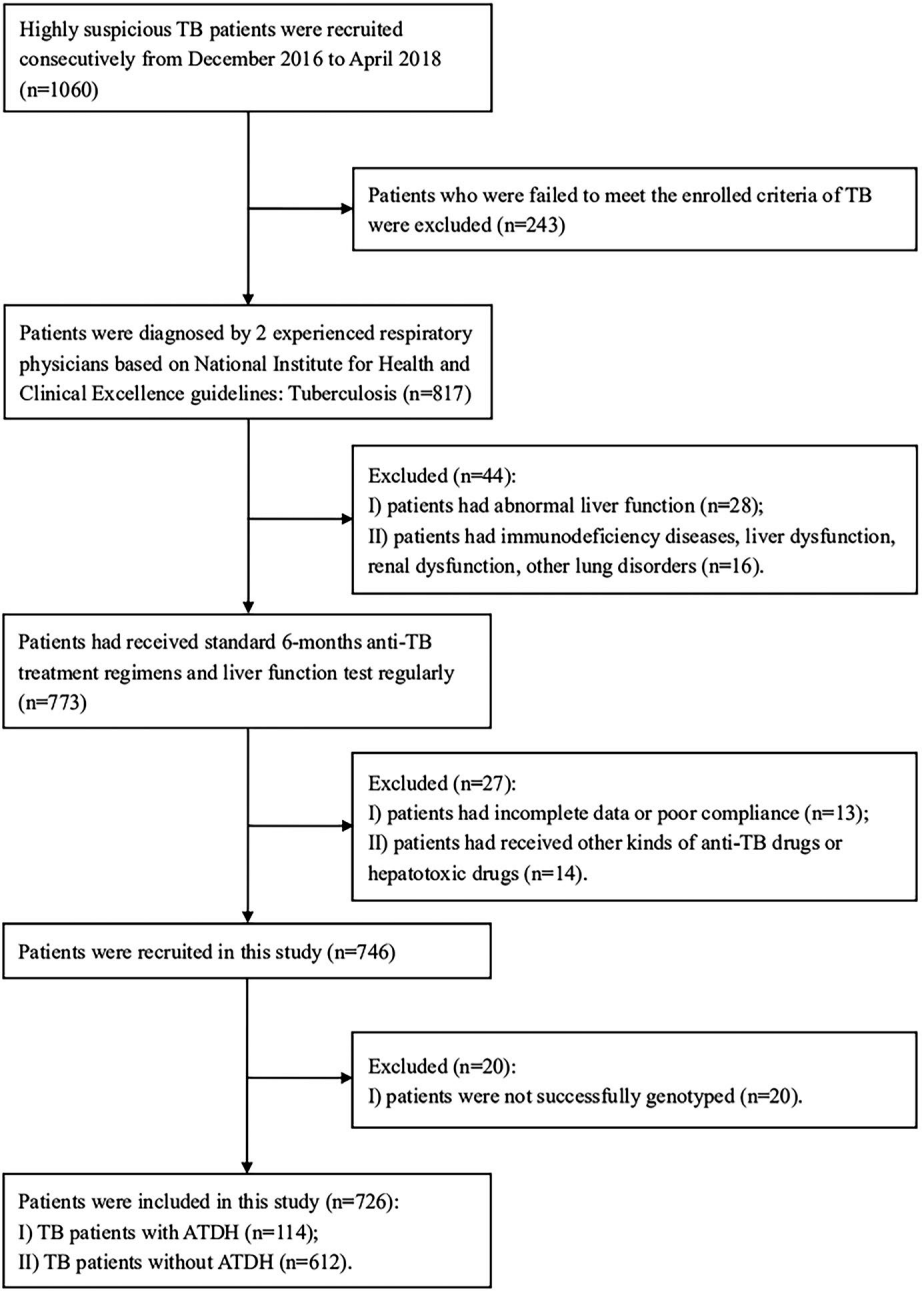
Flow diagram of selection of patients enrolled. Abbreviations: TB: tuberculosis; ATDH: anti‐tuberculosis drug‐induced hepatotoxicity

A total of 746 TB patients were included in our study, while 726 patients were successfully genotyped with all selected SNPs (Figure 1). The incidence rate of ATDH was 15.70% (114/726). No differences in age (*P* = 0.240) and gender (*P* = 0.752) were found between the cases and the controls. Significant differences in the incidence of fever (*P* = 0.007), weight loss (*P* = 0.036), total bilirubin levels (*P* = 0.003), serum ALT levels (*P* < 0.001), serum AST levels (*P* < 0.001), uric acid levels (*P* = 0.019), alkaline phosphatase (ALP) levels (*P* = 0.010), and gamma glutamyl transpeptidase (GGT) levels (*P* < 0.001) were observed between these two groups. The demographic and clinical characteristics of all enrolled 726 patients are depicted in Table S1.

### 3.2 Single selected SNP association with ATDH

All of the 11 SNPs genotypes from the controls did not deviate from the HWE. The mutant A allele frequency of rs12483377 was 0.46% and 3.29% in the cases and the controls, respectively. The mutant A allele conferred the decreased risk of ATDH (OR = 0.13, 95%CI: 0.02–0.98, *P* = 0.020). Logistic regression showed that this significance still existed after adjusting age and gender (*P* = 0.024), and the statistical power is 0.77 (Table 1). No case harbored AA genotype of rs12483377, while there were 2 patients with AA genotype in the controls. For the AG genotype of rs12483377, there was 1 and 35 subjects in the ATDH group and non‐ATDH group, respectively. However, as shown in Table 2, comparable risk of ATDH was identified in these 3 genetic models of rs12483377. As for the genetic models of the other 10 selected SNPs, none of them reach the threshold value of statistical significance.

**Table 1:** The distributions of allelic and genotypic frequency between non‐ATDH^a^ and ATDH^b^ for the selected 11 SNPs

SNP	Allele	Group	Allele					Genotype			P_HWE_
			1^b^/2^b^	OR (95% CI)	*P*	*P* *	Power	11^b^/12^b^/22^b^	*P*	*P* *	
rs2236455	A>G	ATDH^a^	56/172	0.92 (0.67–1.29)	0.653	0.449		9/38/67	0.829	0.543	0.311
		Non‐ATDH^a^	318/906					48/222/342			0.171
rs114220916	G>A	ATDH^a^	15/213	1.11 (0.63–2.00)	0.721	0.821		0/15/99	NA	0.752	1.000
		Non‐ATDH^a^	73/1151					0/73/539			0.157
rs9980080	G>A	ATDH^a^	40/188	1.07 (0.74–1.56)	0.722	0.785		3/34/77	0.635	0.590	1.000
		Non‐ATDH^a^	203/1021					22/159/431			0.143
rs2236467	G>A	ATDH^a^	16/212	1.09 (0.63–1.91)	0.752	0.980		0/16/98	NA	0.790	1.000
		Non‐ATDH^a^	79/1145					0/79/533			0.166
rs13048803	G>A	ATDH^a^	39/189	1.10 (0.75–1.60)	0.635	0.705		2/35/77	0.763	0.578	0.521
		Non‐ATDH^a^	194/1030					13/168/431			0.546
rs2838942	A>G	ATDH^a^	16/212	1.09 (0.63–1.91)	0.752	0.963		0/16/98	NA	0.792	1.000
		Non‐ATDH^a^	79/1145					0/79/533			0.166
rs9980525	G>A	ATDH^a^	39/189	1.12 (0.77–1.64)	0.547	0.631		2/35/77	0.777	0.528	0.521
		Non‐ATDH^a^	190/1034					11/168/533			0.284
rs3753019	G>A	ATDH^a^	108/120	1.11 (0.84–1.48)	0.456	0.455		27/54/33	0.460	0.869	0.578
		Non‐ATDH^a^	547/677					115/317/180			0.253
rs2236483	G>A	ATDH^a^	93/135	0.82 (0.61–1.09)	0.168	0.199		18/57/39	0.322	0.364	0.846
		Non‐ATDH^a^	560/664					134/292/186			0.330
rs12483377	G>A	ATDH^a^	1/227	**0.13 (0.02–0.98)** ^c^	**0.020**	**0.024**	0.77	0/1/113	NA	0.055	1.000
		Non‐ATDH^a^	39/1185					2/35/575			0.119
rs7867	A>G	ATDH^a^	98/130	0.86 (0.65–1.15)	0.32	0.262		23/52/39	0.592	0.298	0.450
		Non‐ATDH^a^	570/654					139/292/181			0.330

Abbreviations: CI, Confidence interval; HWE, Hardy‐Weinberg equilibrium; NA, Non‐available; OR, Odd ratio; SNP, Single nucleotide polymorphisms.

^a^ Non‐ATDH and ATDH refer to patients without and with anti‐tuberculosis drug‐induced hepatotoxicity, respectively;

^b^ “1”: mutant allele; “2”: wild allele; “11”: mutant homozygote; “12”: heterozygote; “22”: wild homozygote;

^c^ Statistically significant data will be highlighted in bold.

* *P* value after adjusting the age and gender.

**Table 2:** Genetic models analyses of the selected 11 SNPs

SNP	Model	OR (95% CI)	*P*	*P* *, a
rs2236455 (A>G)	Add	0.93 (0.68–1.28)	0.663	0.845
	Dom	0.89 (0.59–1.33)	0.568	0.538
	Rec	1.01 (0.48–2.12)	0.985	0.950
rs114220916 (G>A)	Add	1.12 (0.62–2.03)	0.712	NA
	Dom	1.12 (0.62–2.03)	0.712	0.752
	Rec	NA (NA– NA)	NA	NA
rs9980080 (G>A)	Add	1.07 (0.74–1.54)	0.728	0.752
	Dom	1.14 (0.75–1.76)	0.538	0.571
	Rec	0.72 (0.21–2.46)	0.606	0.590
rs2236467 (G>A)	Add	1.10 (0.62–1.97)	0.743	NA
	Dom	1.10 (0.62–1.97)	0.743	0.790
	Rec	NA (NA–NA)	NA	NA
rs13048803 (G>A)	Add	1.10 (0.75–1.62)	0.629	0.859
	Dom	1.14 (0.75–1.76)	0.538	0.571
	Rec	0.82 (0.18–3.70)	0.799	0.819
rs2838942 (A>G)	Add	1.10 (0.62–1.97)	0.743	NA
	Dom	1.10 (0.62–1.97)	0.743	0.792
	Rec	NA (NA–NA)	NA	NA
rs9980525 (G>A)	Add	1.13 (0.77–1.67)	0.537	0.998
	Dom	1.16 (0.76–1.79)	0.492	0.525
	Rec	0.98 (0.21–4.46)	0.975	0.938
rs3753019 (G>A)	Add	1.12 (0.84–1.49)	0.448	0.416
	Dom	1.02 (0.66–1.59)	0.920	0.951
	Rec	1.13 (0.83–2.16)	0.228	0.213
rs2236483 (G>A)	Add	0.82 (0.62–1.09)	0.174	0.140
	Dom	0.84 (0.55–1.23)	0.419	0.420
	Rec	0.67 (0.39–1.15)	0.143	0.132
rs12483377 (G>A)	Add	0.14 (0.02–1.04)	0.054	0.999
	Dom	0.14 (0.02–1.01)	0.051	0.055
	Rec	NA (NA–NA)	NA	0.999
rs7867 (A>G)	Add	0.87 (0.66–1.15)	0.330	0.337
	Dom	0.81 (0.53–1.23)	0.323	0.308
	Rec	0.86 (0.52–1.41)	0.551	0.522

Abbreviations: Add, Addictive model; CI, Confidence interval; Dom, Dominant model; NA, Non‐available; OR, Odd ratio; Rec, Recessive model; SNP, Single nucleotide polymorphisms.

* *P* value after adjusting the age and gender.

### 3.3 Haplotype construction

**Figure 2:**
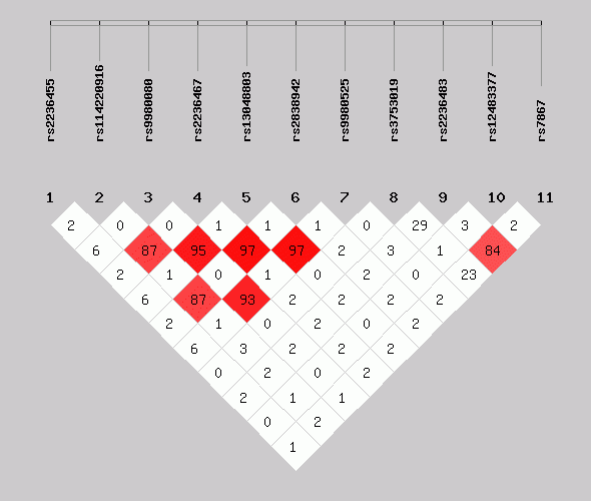
Linkage disequilibrium (LD) map of all single nucleotide polymorphisms (SNPs) in Collagen type XVIII alpha 1 chain (*COL18A1*). The threshold was set at pairwise *r* ^2^ > 0.80. The color of diamonds, paired with the percentages in diamonds, indicates the pairwise *r* ^2^ of all selected SNPs. Namely, the darker the color is, the higher the percentage is

With a threshold of pairwise *r* ^2^ value ≥ 0.80, 3 SNPs (rs114220916, rs9980080, and rs2236467) were in a LD block as well as 3 SNPs (rs2236467, rs13048803, and rs2838942), 5 SNPs (rs114220916, rs9980080, rs2236467, rs13048803, and rs2838942), 5 SNPs (rs9980080, rs2236467, rs13048803, rs2838942, and rs9980525) and 3 SNPs (rs2236483, rs12483377, and rs7867). However, none of the constructed haplotypes of *COL18A1* showed the significant associations with the risk of ATDH (Table 3). The LD plot of selected SNPs are presented in the Figure 2.

**Table 3:** Analysis of haplotypes assigned by *COL18A1* variants with the risk of ATDH^*^, ^a^

Haplotype	OR (95% CI)	*P*	Frequency				Cumulative
			ALL(n = 726)	ATDH^*^, ^a^ (n = 112)	Non‐ATDH^*^, ^a^ (n = 614)	Frequency	
Rs114220916‐ rs2236467	GGG	1.00 (NA–NA)	NA	0.77	0.76	0.78	0.77
	GAG	0.87 (0.59–1.29)	0.49	0.16	0.17	0.16	0.93
	AGA	0.79 (0.42–1.49)	0.47	0.05	0.06	0.05	0.99
Rs2236467‐ rs2838942	GGA	1.00 (NA–NA)	NA	0.77	0.76	0.78	0.77
	GAA	0.89 (0.60–1.31)	0.55	0.16	0.17	0.16	0.93
	AGG	0.87 (0.48–1.57)	0.65	0.06	0.07	0.06	1.00
Rs114220916‐ rs2838942	GGGGA	1.00 (NA–NA)	NA	0.77	0.76	0.78	0.77
	GAGAA	0.86 (0.58–1.28)	0.46	0.16	0.17	0.16	0.93
	AGAGG	0.79 (0.42–1.49)	0.46	0.05	0.06	0.05	0.99
Rs9980080‐ rs9980525	GGGAG	1.00 (NA–NA)	NA	0.77	0.76	0.78	0.77
	AGAAA	0.84 (0.56–1.26)	0.39	0.16	0.17	0.16	0.93
	GAGGG	0.82 (0.45–1.53)	0.54	0.06	0.07	0.06	0.99
Rs2236483‐ rs7867	GGA	1.00 (NA–NA)	NA	0.52	0.55	0.52	0.52
	AGG	1.14 (0.85–1.53)	0.38	0.41	0.39	0.41	0.93
	GGG	0.58 (0.27–1.25)	0.17	0.03	0.04	0.02	0.96
	AAG	6.69 (0.92–48.87)	0.06	0.03	0.00	0.03	0.98
	AGA	0.75 (0.24–2.31)	0.61	0.01	0.02	0.01	1.00

Abbreviations: Add, Addictive model; CI, Confidence interval; NA, Non‐available; OR, Odd ratio.

^a^ Non‐ATDH and ATDH refer to patients without and with anti‐tuberculosis drug‐induced hepatotoxicity, respectively.

### 3.4 The correlation within SNPs and laboratory indicators

**Figure 3:**
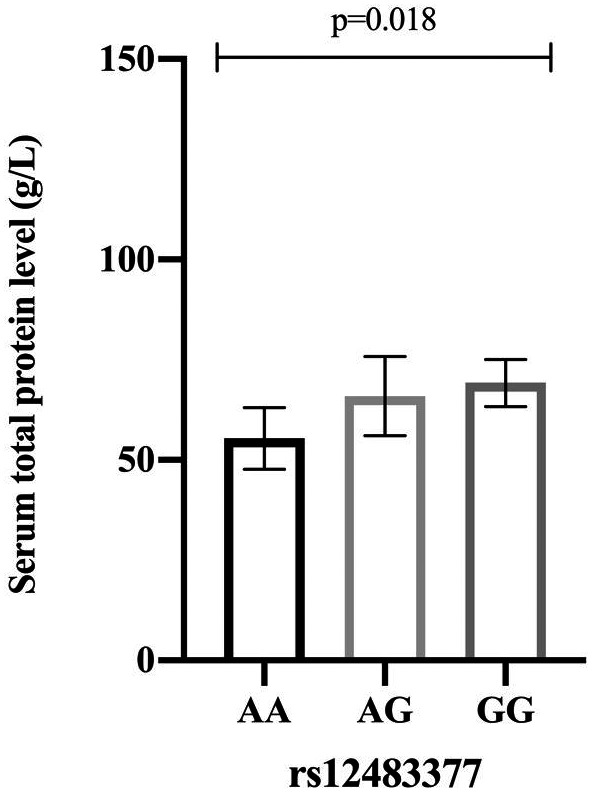
The association between rs12483377 and serum total protein level (g/L) among enrolled patients

For rs12483377, mutant homozygote AA genotype was associated with lower total protein (*P* = 0.018). However, no significant findings on the relationship between rs12483377 and other clinical characteristics were observed (Figure 3). The total protein levels under different genotypes were displayed in Table 4.

**Table 4:** The correlation within rs12483377 and total protein

Genotype	Number	Median (percent 25%–75%)
AA	2	55.40 (51.55–59.25)
AG	36	69.10 (59.70–72.50)
GG	688	69.65 (63.90–75.30)

## 4 DISCUSSION

In this present study, we investigated the relationship between *COL18A1* polymorphisms and the risk of ATDH in the Western Chinese Han population. We revealed that the mutant A allele of *CLO18A1* rs12483377 was associated with decreased risk of ATDH. Furthermore, the statistical significance of rs12483377 on total protein had been identified.

*COL18A1*, highly expressing in the liver (http://biogps.org/#goto=genereport&id=80781), is closely related to liver diseases.^25^, ^42^, ^43^ Musso et al^44^ have reported the relationship between *COL18A1* and liver fibrosis. Type XVIII collagen, encoded by *COL18A1*, can increase before and during the fibrotic stages of liver fibrosis.^45^, ^46^ The increased type XVIII collagen upregulates its own product, EST. EST is able to resist liver fibrosis by inhibiting the expression of TGF‐β1 mRNA through RhoA/ROCK signal pathways in hepatic stellate cells (HSCs).^47^, ^48^ In addition, liver fibrosis refers to the progression of extracellular matrix excessive deposition, which is often promoted by the activation of HSCs. HSCs can transdifferentiate into cells which can secrete extracellular matrix.^49^, ^50^, ^51^ Therefore, *COL18A1* is associated with liver fibrosis via regulating the expression of EST *COL18A1* is also related to hepatic carcinoma by EST.^52^ EST can inhibit endogenous angiogenesis by suppressing the production of angiogenic factors, while angiogenesis is a common physiological and pathological process in liver cancer.^53^, ^54^ Besides, the relationship between *COL18A1* polymorphisms and liver cancer is also reported. Wu et al^36^ have suggested that *COL18A1* rs7499, located in the 3’‐UTR region, increases the risk of hepatocellular carcinoma in Chinese Han population by negatively working in the binding site for has‐mir‐328. Based on these relationships, both *COL18A1* and its product, EST, are considered as targets of liver cancer due to their function of restricting of endothelial proliferation and inhibiting the growth and metastasis of tumors.^55^, ^56^, ^57^ Furthermore, Duncan et al^30^ have revealed that type XVIII collagen is vital to preserve the integrity of liver during hepatotoxic injury through α1β1 integrin, integrin linked kinase and the Akt pathway. *COL18A1* is thus identified as a necessary survival factor of acute liver injury from carbon tetrachloride.

In our study, we firstly reported the relationship between *COL18A1* variants and ATDH susceptibility. We found that rs12483377 is associated with decreased risk of ATDH. It has been verified that the mutant A allele of rs2483377 could decrease the ability of EST to bind other molecules and the function to inhibit angiogenesis.^58^ Considering the role of EST in the occurrence of ATDH as we described before, we deduced that rs12483377 may influence ATDH by regulating the expression of EST which participates in oxidative stress.^20^, ^26^, ^27^ In addition, it is well‐recognized that expression quantitative trait locus (eQTL) regulates the expression level of mRNA and protein specifically, and the expression level of mRNA/protein is proportional to the quantitative character.^59^, ^60^ Rs12483377 has 2 hits of cis eQTL hits (http://pubs.broadinstitute.org/mammals/haploreg/detail_v4.1.php?query=&id=rs12483377). It is reported that rs12483377 involved in regulating the expression of both *COL18A1* and solute carrier family 19 member 1 (*SLC19A1*).^61^, ^62^ Thus, we speculated that rs12483377 might reduce the expression of EST through eQTL, and thus functioned in the occurrence of ATDH.

Besides, in our study, there were significant differences in fever, weight loss, total bilirubin levels, serum ALT levels, serum AST levels, uric acid levels, ALP levels, and GGT levels between the case and control group. Fever and weight loss are the common symptoms of tuberculosis poisoning.^63^ After Mycobacterium tuberculosis infected the body, it will produce toxins and metabolites, which can not only cause allergic reactions such as fever, fatigue, and so on, but also will stimulate the central nervous system, resulting in dysfunction of the autonomic nervous system which lead to night sweats.^63^, ^64^ Bilirubin, ALT, AST, ALP, and GGT are all associated with the liver metabolism.^65^, ^66^ As is stated before, anti‐TB treatment can result in liver injury through four mechanisms.^3^ As for uric acid levels, all patients enrolled were treated with INH, RIF, PZA, and EMB, in which INH, PZA, EMB, and their metabolites could compete with uric acid for the organic acid excretion pathway, reducing the excretion of uric acid, thus causing the increase of uric acid y.^67^ In this study, a statistical significance on the relationship between mutant homozygote AA and total protein was found. However, after reviewing the relevant literature, it is found that the current reports are not enough to explain the two situations, so it may just be a statistical correlation and may not be of any clinical significance. Meanwhile, after reviewing the specific circumstances of these cases, the impact of the low number of homozygote AA on the analysis in this study can not be ruled out. Furthermore, this may also be an innovative finding, so the recruitment of people for the function verification and larger population verification and so on aiming for rs12483377 is under way.

### 4.1 Strengths and limitations

We firstly investigated the relationship between *COL18A1* polymorphisms and ATDH susceptibility in Western Chinese Han population. Based on available evidence, we also speculated the potential mechanisms that how *COL18A1* polymorphisms affect ATDH susceptibility, contributing to the deep understanding of ATDH etiology to some extent. Besides, our finding is beneficial to explore more novel biomarkers of ATDH and decrease the burden brought by ATDH to some degree. Nevertheless, there were still some limitations in our study. The design of single center study restricts us to verify our findings in different ethnicities. Functional experiment about rs12483377 should have been further performed to validate our speculation. More high‐quality studies with lager cohorts are warranted.

## 5 CONCLUSION

Collectively, our study revealed that *CLO18A1* rs12483377 is related to the risk and specific characteristic of ATDH in the Western Chinese Han population, mining and further emphasizing the role of *CLO18A1* variants in ATDH.

## CONFLICT OF INTEREST

Author Yuhui Cheng, Author Lin Jiao, Author Weixiu Li, Author Jialing Wang, Author Zhangyu Lin, Author Hongli Lai, Author Binwu Ying declare that they have no conflict of interest.

## AUTHORS CONTRIBUTIONS

Research design: Binwu Ying. Data collection: Yuhui Cheng, Lin Jiao, Weixiu Li, Jialing Wang, Zhangyu Lin. Data analysis: Yuhui Cheng, Lin Jiao, Weixiu Li, Jialing Wang, Zhangyu Lin, Hongli Lai. Project administration: Binwu Ying. Writing‐original draft: All authors. Writing‐revision: All authors.

## Supporting information

Table S1

## Data Availability

All data to this article can be found at the end of this manuscript.
