# Supplementary material for: Collagen type XVIII alpha 1 chain (COL18A1) variants affect the risk of anti‐tuberculosis drug‐induced hepatotoxicity: A prospective study
Source: J Clin Lab Anal. 2020 Dec 9;35(2):e23630. doi: 10.1002/jcla.23630 (PMC7891502; doi:10.1002/jcla.23630)
Supplement: Supplementary file 1 — Table S1 [file JCLA-35-e23630-s001.docx]

**Table 1** The demographic, clinical characteristics and laboratory examinations of enrolled patients.

| **Characteristics** | **Non-ATDH ^*^ (n=612)** | **ATDH ^*^ (n=114)** | **P-value** |
| --- | --- | --- | --- |
| **General information** | | | |
| Age, years, (mean±standard) | 42.98±18.47 | 40.49±15.64 | 0.240 |
| Sex (male/female) | 364/248 | 66/48 | 0.752 |
| Smoking, no/yes | 395/217 | 78/36 | 0.425 |
| Drinking, no/yes | 455/157 | 81/33 | 0.463 |
| **Clinical symptoms** | | | |
| Fever, n (%) | 276 (45.10) | 67 (58.77) | **0.007** |
| Weight loss, n (%) | 252 (41.18) | 35 (30.70) | **0.036** |
| Night sweat, n (%) | 189 (30.88) | 31 (27.19) | 0.431 |
| Poor appetite, n (%) | 244 (39.87) | 48 (42.11) | 0.655 |
| Fatigue, n (%) | 163 (26.63) | 32 (28.07) | 0.751 |
| **Laboratory indicators, median (percent25%-75%)** | | | |
| Erythrocyte (*10^12^/L) | 4.32 (3.78-4.74) | 4.28 (3.99-4.77) | 0.572 |
| Hemoglobin (g/L) | 123.00 (106.25-137.00) | 123.00 (109.00-138.00) | 0.592 |
| Hematocrit (L/L) | 0.38 (0.32-0.41) | 0.37 (0.34-0.42) | 0.171 |
| Platelet (*10^9^/L) | 233.00 (171.25-296.00) | 236.00 (185.00-321.00) | 0.132 |
| WBC ^a^ (*10^9^/L) | 6.42 (5.08-8.34) | 6.61 (5.05-7.97) | 0.889 |
| Neutrophil (%) | 71.35 (62.73-78.70) | 71.00 (63.90-78.90) | 0.818 |
| Leucocyte (%) | 18.20 (12.70-26.00) | 17.50 (12.50-26.20) | 0.624 |
| Monocyte (%) | 7.10 (5.70-8.80) | 7.60 (5.60-9.40) | 0.158 |
| CRP ^b^ (mg/L) | 12.60 (2.60-38.80) | 11.80 (3.12-42.68) | 0.942 |
| ESR^c^(mm/h) | 31.00 (14.00-63.00) | 41.00 (20.50-63.25) | 0.137 |
| TBIL^d^ (μmol/L) | 8.70 (6.40-12.20) | 10.05 (7.50-14.53) | **0.003** |
| DBIL ^e^ (μmol/L) | 3.50 (2.50-5.30) | 3.80 (2.48-6.85) | 0.085 |
| IBIL ^f^ (μmol/L) | 5.00 (3.40-7.10) | 5.60 (3.78-7.48) | 0.070 |
| ALT ^g^ (IU/L) | 15.00 (10.00-21.00) | 26.00 (15.00-38.00) | **<0.001** |
| AST ^h^ (IU/L) | 20.00 (16.00-25.00) | 26.00 (19.00-33.00) | **<0.001** |
| TP ^i^ (g/L) | 69.30 (63.20-75.00) | 70.00 (63.90-75.40) | 0.503 |
| Albumin (g/L) | 38.70 (33.10-43.18) | 38.50 (34.25-43.98) | 0.285 |
| Globulin (g/L) | 30.20 (26.00-34.60) | 30.20 (26.15-35.03) | 0.881 |
| Glucose (mmol/L) | 5.14 (4.71-5.86) | 5.07 (4.62-5.90) | 0.460 |
| Urea (mmol/L) | 4.09 (3.13-5.30) | 3.99 (2.91-5.22) | 0.349 |
| Creatinine (μmol/L) | 60.00 (49.00-73.00) | 59.60 (49.75-70.30) | 0.711 |
| Cystatin c (mg/L) | 0.92 (0.79-1.06) | 0.90 (0.80-1.06) | 0.953 |
| Uric acid (μmol/L) | 301.00 (224.00-408.00) | 272.00 (197.95-381.00) | **0.019** |
| Triglyceride (mmol/L) | 1.02 (0.77-1.39) | 0.97 (0.82-1.29) | 0.659 |
| Cholesterol (mmol/L) | 3.82 (3.15-4.61) | 3.84 (3.12-4.65) | 0.985 |
| HDL-C ^j^ (mmol/L) | 1.09 (0.83-1.43) | 1.12 (0.85-1.48) | 0.531 |
| LDL-C^k^ (mmol/L) | 2.20 (1.69-2.75) | 2.17 (1.66-2.71) | 0.844 |
| ALP ^l^ (IU/L) | 79.50 (64.00-97.00) | 84.50 (70.50-105.50) | **0.010** |
| GGT ^m^ (IU/L) | 29.00 (18.00-48.00) | 42.50 (26.00-78.25) | **<0.001** |

**Notes:** ^*^: Non-ATDH and ATDH refer to patients without and with anti-tuberculosis drug induced hepatotoxicity, respectively.

**Abbreviations:** a: white blood cell; b: C-reactive protein; c: erythrocyte sedimentation rate; d: total bilirubin; e: direct bilirubin; f: indirect bilirubin; g: alanine aminotransferase; h: aspartate transaminase; i: total protein; j: high density lipoprotein cholesterol; k: low density lipoprotein cholesterol; l: alkaline phosphatase; m: gamma glutamyl transpeptidase.
